# Effects of Occupational and Leisure-Time Physical Activities on Insomnia in Korean Firefighters

**DOI:** 10.3390/ijerph17155397

**Published:** 2020-07-27

**Authors:** Myeongseob Lim, Kyoung Sook Jeong, Sung-Soo Oh, Sang-Baek Koh, Sei-Jin Chang, Yeon-Soon Ahn

**Affiliations:** 1 Department of Occupational and Environmental Medicine, Wonju Severance Christian Hospital, Yonsei Wonju College of Medicine, Yonsei University, Wonju 26426, Korea; kwe4501@naver.com (M.L.); bandyoem@naver.com (K.S.J.); oss0609@yonsei.ac.kr (S.-S.O.); 2 Department of Preventive Medicine and Genomic Cohort Institute, Yonsei Wonju College of Medicine, Yonsei University, Wonju 26426, Korea; kohhj@yonsei.ac.kr; 3 Department of Preventive Medicine, Institute of Occupational and Environmental Medicine, Yonsei Wonju College of Medicine, Yonsei University, Wonju 26426, Korea; chang0343@yonsei.ac.kr

**Keywords:** firefighters, physical activity, occupational physical activity, leisure time physical activity, job loading, insomnia

## Abstract

Studies have been conducted on the association between physical activity (PA) and sleep, but to the best of our knowledge, a simultaneous analysis of the effects of occupational PA (OPA) and leisure time PA (LTPA) on South Korean firefighters’ sleep has never been conducted. This study aims to analyze how OPA and LTPA affect these individuals’ risk of suffering from insomnia with-in this specific population of subjects. The study includes data from an online self-report survey in which 9788 South Korean firefighters participated. The survey used the Insomnia Severity Index and the OPA- and LTPA-related characteristics were investigated. The independent two-sample *t*-test, χ^2^ test, and multiple logistic regression analysis were performed. Subgroup logistic regression analyses were also completed in accordance with the OPA level. Among 9788 participants, 890 (9.1%) suffered from insomnia. A logistic regression analysis revealed that higher levels of feeling of job loading (FoJL), rising levels of physical strength utilization rate (PSUR), greater frequency levels of occupational activities, and high-intensity LTPA were significantly correlated with an increased risk of insomnia, while execution of LTPA and getting enough rest after LTPA was correlated with a decreased risk. However, the subgroup analysis showed that high-intensity LTPA was correlated with a significantly increased the risk in the group with high OPA, but this did not apply to the group with low OPA. Although the risk of suffering from insomnia was overall significantly higher in the high OPA group, the risk was significantly lower in groups getting enough rest after partaking in LTPA, regardless of the OPA level. Thus, the intensity of exercise programs pre-scribed to groups with high OPA and individuals with higher risks of suffering from insomnia, such as firefighters, police officers, and soldiers, should be considered.

## 1. Introduction

Insomnia is a common sleeping disorder in the general population [1] and is associated with several sociodemographic and psychological factors. Previous studies have suggested that gender, levels of education or socioeconomic status, marital status, smoking and alcohol consumption, caffeine intake, and psychiatric comorbidities, such as depression and anxiety, are associated with insomnia. More specifically, these past studies indicate that being female, having a lower level of education and socioeconomic status, greater alcohol consumption and caffeine intake, and simultaneously suffering from other psychological disorders are strongly associated with insomnia [2,3,4,5].

It is important to address how physical activities impact individuals’ sleep and there have been studies reporting positive effects of physical activity (PA) on sleep [6]. It has been noted that people who are physically active complain less about their sleep problems [7,8]. Furthermore, a previous study suggested that PA itself can be used as a non-pharmacological treatment for sleep disorders [9]. Although the effects of PA on sleep and insomnia symptoms are controversial as it depends on the type, time, or intensity of the specific PA [3,10]; in general, PA seems to have a more positive effect on sleep.

However, the PA mentioned in these studies is restricted to leisure time PA (LTPA). In addition to LTPA, another important category of PA that must be considered is occupational PA (OPA) [11]. OPA refers to all work-related demands exerted on workers, not only mechanical loads, such as long working hours, but also metabolic loads and feelings of burden [12,13]. Unlike LTPA, OPA is strongly associated with negative health conditions [14]. Regarding sleep, Skarpsno et al. measured the OPA and LTPA of individuals, using an accelerometer, and reported that LTPA was associated with a low prevalence of sleep problems, while OPA was associated with a high prevalence [11]. Thus, because OPA and LTPA may have contrasting effects on individual’s sleep, it is necessary to examine them separately.

Focusing on a more specific population of individuals, it is noted that firefighters have a higher level of OPA than other occupational groups [15]. According to the U.S. Department of Labor, the basic tasks of firefighters, such as ladder climbing, fire suppression, lifesaving, and carrying hoses and heavy loads, are labelled as “heavy” or “very heavy,” and are mostly over 6.8 METs (Metabolic Equivalent of Task) [16,17]. For firefighters specifically in South Korea (hereafter referred to as Korea), the basic tasks of their occupation, such as forced smoke ventilation, hose deployment, and lifesaving during firefighting were also evaluated as “very heavy” tasks [18]. Relating this population group to the topic of sleep problems, it has been reported that firefighters have a higher prevalence of sleep disorders [19]. However, because only a few studies on sleep disorders have been evaluated by ISI (insomnia severity index), making a clear comparison can be difficult.

To the extent of our knowledge, no prior studies have examined the association between firefighters’ PA (OPA, LTPA, or both) and their risk of suffering from insomnia. It was reported that the risk of suffering from insomnia was more frequent [20] in shift-work firefighters compared to day-work firefighters. Furthermore, there was a strong association between the frequency of emergency and off-duty work and insomnia in Korean firefighters [21]. However, these studies only included OPA in their analysis. As far as the authors know, there has been no studies conducted on the association between insomnia and OPA and LTPA in groups with high OPA (i.e., firefighters, soldiers, and police officers).

The purpose of this study is to analyze how firefighters’ OPA and LTPA affect their risk of suffering from insomnia as it provides the basic data recommending the appropriate LTPA level for firefighters, a group with a high level of OPA.

## 2. Materials and Methods 

### 2.1. Subjects

The subjects were recruited through an online web survey conducted from November 2017 to August 2018. Initially, a total of 9811 subjects were recruited, but after excluding 23 subjects who did not respond to any of the questions (e.g., basic sociodemographic variables), there were a total of 9788 subjects who were officially included in the study. Prior to the survey, the purpose of the study was explained, and it was stated that all data would be anonymously processed and used solely for the purpose of policy-making and research of the firefighting activity system. The study was approved by the Institutional Review Board of Yonsei University Wonju Severance Christian Hospital (CR318031).

### 2.2. Subjective Occupational Physical Activity

To evaluate the subjective OPA, we investigated the feeling of job loading (FoJL) (“How physically burdening is your work?”) and the physical strength utilization rate (PSUR) during work (“How much of your maximum physical strength do you use during work?”). These questions were developed as reference by the authors.

To measure the subjects’ FoJL, they were given four options to choose from: “none,” “a little bit hard,” “moderately hard,” or “very hard.” The FoJL was then divided into two broader groups as “none or little,” which referred to the subjects who responded as “none” and “a little bit hard,” or “moderate to high” which referred to those who responded as “moderately hard” and “very hard.”

The subjects were also given four categories to choose from for PSUR: <50%, ≥50% and <80%, ≥80% and <100%, and ≥100%. Once again, we divided them into two broader groups: <80% (reference) or ≥80%.

We operationally defined the subjective OPA by combining the two variables. The subjective OPA was evaluated as “low” when the subjects responded to the FoJL as “none or little” and their PSUR < 80%, while the rest were evaluated as “high” subjective OPA. The criteria were established arbitrarily by the authors.

### 2.3. Objective Occupational Physical Activity

To evaluate the objective OPA, we investigated the work schedule (“Are you currently working shifts?”), the frequency of the occupational activities (“In the last 6 months, how many times a week, on average, did you go to work in your department?”), and the frequency of off-duty work (“How many times a month do you work on off-duty days due to manpower vacancy?”).

The work schedule was labelled as “day work” or “shift work.” The frequency of occupational activities implied the number of cases per week and was classified as <5 (reference) or ≥5 times a week. The frequency of off-duty work was evaluated in the form of nominal variables rather than continuous variables: “less than once a month,” “once or twice a month,” “3 or 4 times a month,” or “≥5 times a month.” We then classified them in a broader group as <3 (reference) or ≥3 times a month.

We defined the objective OPA operationally by combining the work schedule, the frequency of occupational activities, and the frequency of off-duty work. In the case of day work, it was evaluated as “low” objective OPA. Furthermore, even in the case of shift work, if the frequency of the occupational activities was <5 times a week and the frequency of off-duty work was also <3 times a month, it was considered to be low-objective OPA. Other cases were evaluated as high-objective OPA.

### 2.4. Leisure Time Physical Activity

The LTPA was assessed via the following yes or no question: “Are you currently training to improve your physical fitness or health?” Only the subjects who answered yes were surveyed for the type of LTPA, intensity of LTPA, and the sufficient resting period after LTPA. 

The different categories of LTPA included “aerobic,” “anaerobic,” or “both,” and the subjects’ response was divided into those who “do not perform aerobic PA (anaerobic only, reference)” or those who do “perform aerobic PA (aerobic only and both).” 

The subjects were then asked to answer how many times they partake in LTPA a week and how many minutes the LTPA lasts per session. They were also asked to indicate the intensity level of the PA with “high intensity,” “medium intensity,” or “low intensity.” The criteria for the intensity level of the PA were specified in the questionnaire in the following manner: High intensity implies the PA causes shortness of breath or a higher heart rate, whereas medium intensity implies the PA causes slight shortness of breath or slightly elevated heart rate. We re-assessed the LTPA intensity by combining the frequency, the time length of each session, and the intensity of the PA as provided by the subjects. Operationally, the LTPA intensity was defined as high for the subjects who spend more than five times a week doing a 30-min medium-intensity PA per session or those who spend three times a week doing 30-min-high-intensity PA. These criteria were established with reference to the Physical activity guidelines for Americans [22]. 

Lastly, the sufficient resting period after LTPA was evaluated with the following yes or no question: “Do you get enough rest to recover after LTPA?” 

### 2.5. Severity of Insomnia

Insomnia severity level was assessed using the ISI [23]. This index consists of seven items that evaluate insomnia severity over the past 2 weeks. ISI scores range from 0 to 28, assessing the severity of insomnia symptoms as none with a score ≤ 7, mild with a score between 8 and 14, moderate with a score between 15 and 21, and severe with a score between 22 and 28. Subjects were then categorized as being “normal” or having “insomnia” according to the sum of the ISI scores (<15 or ≥15 respectively).

### 2.6. Assessment of Covariates

Sociodemographic characteristics included age, sex, body mass index (BMI), education, marital status, monthly income, smoking status, alcohol consumption, caffeine intake, and occupation. 

Organizational characteristics included the flexibility of the work schedule and the adequate notice time in cases of schedule change. The work schedule was labelled as flexible if it could be changed freely depending on circumstances or was labelled as non-flexible if it could not be changed. Additionally, the notice time was considered to be inadequate if the notice of schedule change was irregular or occurred on the day of or the day before. 

Psychological characteristics included depression, anxiety, and post-traumatic stress disorder (PTSD). Depression and anxiety were evaluated by using the Patient Health Questionnaire-9 (PHQ-9) [24] and the Generalized Anxiety Disorder-7 (GAD-7) [25], respectively. The presence or absence of these symptoms was assessed based on a scale of 10 points (<10 or ≥10). PTSD was assessed using the Primary Care-PTSD Screen (PC-PTSD) for Diagnostic and Statistical Manual of Mental Disorders-5 (DSM-5) [26]. The PC-PTSD consists of four items that asked the subjects about the presence or absence of PTSD-related symptoms over the past 4 weeks. If more than three items were answered as yes, then PTSD can be suspected.

### 2.7. Statistical Analysis

First, we used independent two-sample *t*-tests and χ^2^ tests to evaluate the difference in the distribution of insomnia according to each covariates and independent variables. Second, logistic regression analysis were conducted to examine the relationship between OPA, LTPA, and insomnia after adjusting the covariates. Finally, we attempted to determine how the effects of LTPA-related characteristics on insomnia differ depending on whether the OPA level is low or high. For this, we obtained the odds of LTPA-related characteristics for insomnia through logistic regression analyses based on whether the subjective OPA and objective OPA levels are low or high. 

Among the covariates, sex, age, education, marital status, monthly income, smoking status, alcohol consumption, caffeine intake, occupation, work schedule flexibility, adequacy of notice time, depression, anxiety, and PTSD were all used as adjusting variables. 

We used the PASW SPSS version 25 (IBM Inc., Armonk, NY, USA) program for statistical analyses and set the significance level to <0.05.

### 2.8. Subgroup Analysis

Because the effect of psychological symptoms is a major confounding factor that could affect insomnia, we performed the logistic regression analysis once more for only subjects without any psychological symptoms in order to exclude this confounding effect.

## 3. Results

### 3.1. Descriptive Statistics 

General characteristics of this study are presented in Table 1. Out of all 9788 participants, 890 (9.1%) were evaluated as suffering from insomnia. There was a statistically significant difference in the average age level according to the presence or absence of insomnia. Furthermore, there was a significant difference in the distribution of insomnia prevalence according to sex, marital status, monthly income, alcohol consumption, caffeine intake, occupation, work schedule flexibility, adequacy of the notice time, and psychological characteristics.

Table 2 shows the distribution of insomnia severity according to the PA-related characteristics. There were significant differences in the distribution of insomnia severity depending on the FoJL, PSUR, frequency of occupational activities, frequency of off-duty work, execution of LTPA, intensity of LTPA, and sufficient resting period after LTPA. However, the difference in the distribution of insomnia severity depending on the work schedule or the type of LTPA was not significant.

### 3.2. Effects of Physical-Activity-Related Characteristics on Insomnia Severity

Table 3 shows the association between PA-related characteristics and the severity of insomnia. Adjusted OR was calculated after adjusting these variables: sex, age, education, marital status, monthly income, smoking status, alcohol consumption, caffeine intake, type of job (office work vs. others), work schedule flexibility, adequacy of the notice time, depression, anxiety, and PTSD. The subjective OPA significantly increased the risk of an individual suffering from insomnia, but the objective OPA was not significantly associated with the severity of insomnia after the adjustment (adjusted OR = 2.12, 95% CI = 1.73–2.59; adjusted OR = 1.19, 95% CI = 0.99–1.42).

Among LTPA-related characteristics, partaking in LTPA and getting enough rest after LTPA correlated with a significantly decreased risk of insomnia, while doing high-intensity LTPA correlated with an increased risk after adjusting the above-mentioned covariates (adjusted OR = 0.73, 95% CI = 0.61–0.88; adjusted OR = 0.40, 95% CI = 0.33–0.50; adjusted OR = 1.37, 95% CI = 1.13–1.67, respectively). The type of LTPA was not significantly associated with insomnia (adjusted OR = 0.88, 95% CI = 0.66–1.16).

### 3.3. Association between LTPA-Related Characteristics and Insomnia Severity According to the OPA Level

Table 4; Table 5 show the association between LTPA-related characteristics and insomnia according to the degree of subjective and objective OPA, respectively.

Regardless of the level of subjective OPA, partaking in LTPA (low subjective OPA: adjusted OR = 0.61, 95% CI = 0.40–0.94; high subjective OPA: adjusted OR = 0.77, 95% CI = 0.62–0.94) and getting enough rest after LTPA (low subjective OPA: adjusted OR = 0.33, 95% CI = 0.20–0.56; high subjective OPA: adjusted OR = 0.43, 95% CI = 0.34–0.55) significantly decreased the risk of suffering from insomnia. In groups with low subjective OPA, performing high-intensity LTPA was not significantly associated with the risk (adjusted OR = 0.96, 95% CI = 0.59–1.56). However, in groups with high subjective OPA, performing high-intensity LTPA significantly increased the risk (adjusted OR = 1.52, 95% CI = 1.23–1.89), unlike the cases of low subjective OPA (Table 4).

The trend found by analyzing the data according to the degree of the objective OPA was similar to that of the found by analyzing according to the subjective OPA. Regardless of the level of the objective OPA, getting enough rest after LTPA significantly decreased the risk of suffering from insomnia (low objective OPA: adjusted OR = 0.40, 95% CI = 0.26–0.62; high objective OPA: adjusted OR = 0.41, 95% CI = 0.32–0.52). Execution of LTPA, the type of LTPA, and the intensity of LTPA was not significantly associated with the risk of suffering from insomnia for the group with low objective OPA. However, in the group with high objective OPA, execution of LTPA significantly decreased the risk (adjusted OR = 0.72, 95% CI = 0.58–0.90), while doing high-intensity LTPA significantly increased the risk of insomnia (adjusted OR = 1.44, 95% CI = 1.15–1.81) (Table 5).

## 4. Discussion

We tried to determine how OPA and LTPA affect the insomnia of Korean firefighters and to provide basic data recommending an appropriate LTPA level for those with high-level OPA. Taking the results of this study into account, in the group with low OPA, considering the firefighters’ work characteristics, doing high-intensity LTPA should be encouraged, but exercise programs should be planned and recommend providing enough rest after exercise. Furthermore, in the group with high OPA, LTPA should be encouraged, while recommending an appropriate LTPA intensity according to one’s physical fitness. Furthermore, getting enough rest after LTPA should be encouraged as well.

OPA is a term that refers to any PA of workers in working environments, metabolic and physical burdens imposed on workers, and the job loading felt about the physical burdens imposed on workers [12,13]. Therefore, in this study, reflecting this point, the FoJL and PSUR during work through the survey responses were also considered OPA and included in the analysis. In previous studies on PA and insomnia, PA was measured objectively using an accelerometer [11,13]. However, since the previous studies were conducted on general population groups, and this study was conducted on firefighters who were already known to have high OPA, it was considered appropriate to apply the above operational definitions.

In our study, we investigated OPA in firefighters doing office work, as well as firefighters working on field (e.g., fire suppression, EMS/rescue, and others). OPA is any PA that takes place during work or in the workplace [27,28,29]. It is easy to think of OPA as a target variable for only occupations requiring considerable physical strength, but recently, several studies have also measured and analyzed OPA among office workers [28,29,30]. For example, Zimring et al. classified the handling of heavy loads or stair climbing in the building as moderate-to-vigorous OPA of office workers [29]. These points can also be applied to firefighters who work in an office. These fire-fighters also face physical demands, such as inspecting firefighting equipment, and on-site support in situations that are undermanned or when large fires occur. In addition, according to a previous study, 24.3% of male and 20.0% of female firefighters doing office work reported that they were exposed to handling heavy loads during work [31]. This means that office work fire-fighters are also exposed to significant OPA.

OPA was divided into subjective and objective OPA. The subjective OPA was intended to reflect how the subjects felt about the physical burden during the work by combining information on FoJL and PSUR during work. The objective OPA was intended to reflect the physical burden that can be quantified by combining objectively measurable variables such as the frequency of occupational activities and off-duty work. The overall trend was similar no matter what type of OPA was considered (objective or subjective). With this in view, we think that the subjective OPA is also meaningful and that using these variables in future studies would be worthwhile. 

As a result of this study, when LTPA was not considered, insomnia prevalence was higher in the group with high OPA than in the group with low OPA. In addition, the insomnia risk was significantly increased in the case of feeling much of physical burden during firefighting work (high subjective OPA) and if the frequency of occupational activities was ≥5 times a week. These results are consistent with previous studies according to which a high level of OPA and working long hours significantly increased the sleep disturbance risk [11,21,32].

Previous studies have shown that the shiftwork itself is not only associated with high OPA [21,32], but can also cause insomnia by disrupting the circadian rhythm [33]. However, the results of this study show that shift work does not significantly affect insomnia. When analyzed without adjusting covariates, shift work was positively associated with insomnia (although not significantly). However, overall results show that there seems to be no association with insomnia since shift work was negatively associated after being adjusted for covariates. This is probably because other factors, such as organizational characteristics, OPA-, and LTPA-related characteristics, had a greater effect on insomnia than shift work itself. The frequency of off-duty work also showed no significant association with insomnia after being adjusted. The reason for this may be that the analysis was based only on the frequency being more than or less than 3 times a month. In fact, Jang et al. reported that the sleep disturbance risk was higher in firefighters whose frequency of off-duty work was 1-2 times a month, 3-4 times a month, or ≥5 times a month, compared to fire-fighters whose frequency of off-duty work was <1 a month, and reported that the result of a trend analysis was also significant [21]. If the frequency of off-duty work had been analyzed in more detail or as a continuous variable in this study, other results would have been derived. Another possible explanation may be that physical burden felt by individuals on workload contributes more significantly to insomnia than the objective workload. Further studies are required to determine which indicators better predict insomnia or are more associated with insomnia, such as subjective burden felt due to work, quantitative load of work such as the frequency of occupational activities, or work intensity elaborately measured by METs.

In the case of the LTPA intensity, the criteria for high-intensity LTPA was ≥5 sessions a week and 30 min or more per session with medium-intensity PA (an activity that causes slight shortness of breath or slightly increased heart rate), or ≥3 sessions a week and 30 min or more per session with high-intensity PA (an activity that causes severe shortness of breath or high heart rate). These criteria are based on the PA guidelines for American adults [22] and were used in other recent studies on voluntary exercise for Korean firefighters [34,35]. However, although this classification system of the LTPA intensity has been used in other studies, it is necessary to measure the exact LTPA intensity by calculating MET, and so on, in further studies.

A few studies have reported that the high-intensity LTPA significantly decreases the insomnia risk [11,36]. However, they considered the effect of only LTPA (not OPA) on insomnia. Fire-fighters are already exposed to a very high level of OPA since they have to wait for an occupational activity such as large-scale fires or accidents at any time, even in during an off-duty day [21]. Considering these, we think that the results of this study were somewhat different from previous studies that targeted the general population.

According to previous studies, the time of day of LTPA, the type of LTPA (whether acute or regular), and resting time after LTPA were factors affecting insomnia. So far, several studies have reported that a vigorous LTPA before few hours of bedtime could affect sleep negatively [8,10]. Furthermore, the effect of when to do LTPA during the day upon insomnia also seems to be controversial [9], but Buxton et al. [37] noted that doing LTPA late in the evening can affect the melatonin secretion, slowing the circadian rhythm and causing insomnia. Resting time after LTPA is also an important factor affecting insomnia. In this related mechanism, serotonin plays an important role, promoting sleep by facilitating the synthesis of hypnogenic compounds, although its effects are various depending on the subtypes [38]. However, in the case of some elite athletes, who often continue to overexert themselves without adequate resting time, a significant decrease in the serotonin level in several brain areas was observed [39]. However, the factors mentioned above were not included or accurately investigated in this study. Therefore, the effect of those factors on insomnia was not analyzed. Further studies on the effects of these factors on insomnia are required.

The presence of depression, anxiety, and PTSD symptoms were adjusted for regression analyses. However, it is known that there is a bidirectional association between insomnia and psychological symptoms [40]. Therefore, to clarify the effects of PA-related characteristics on the risk of insomnia, the same analyses were conducted only for those who did not have psychological symptoms. As a result of the analysis, execution of LTPA and getting enough rest after LTPA significantly decreased the risk of insomnia. Furthermore, in the group with high OPA, doing high-intensity LTPA significantly increased the risk of insomnia. The overall trends of this additional analysis were similar to those of our main finding (Supplementary Materials Tables S1 and S2). In other words, even in groups without psychological symptoms, this study confirmed that OPA and LTPA affect insomnia.

In addition to PA-related characteristics, we tried to control other characteristics that affect insomnia. The results of this study showed that the insomnia prevalence was higher in moderate to heavy drinkers, and in field work firefighters. These results were consistent with the results of previous studies showing the higher the dependence on drinking, the higher the insomnia prevalence [15], and that insomnia prevalence was higher in field work firefighters [21]. Furthermore, insomnia was more common in the group of the subjects who cannot change their work schedule freely or who are noticed of the schedule change on the day or the day before. This coincides with the findings of previous studies that those who have few opportunities to influence the duration of work time, who could not set duty-off days freely, or whose schedule changed frequently are at a high risk of sleep disturbance [32,41,42,43]. These points may apply similarly to our study subjects.

This study has several limitations. First, because of its cross-sectional design, we could not clearly identify the causal relationship between the factors we investigated and the severity of insomnia. Second, subjective OPA was defined, composed, and categorized operationally. This is relevant only in this study and may not be applicable to other studies. However, considering that the tendency between the results according to subjective OPA and the results according to objective OPA is similar, we think that the method of evaluating subjective OPA in this study could be applied to further studies of OPA. Third, since the main outcome of this study, insomnia, was evaluated through a self-report questionnaire, non-response bias and recall bias cannot be excluded. A study of sleep and PA objectified the sleep parameters, such as total sleep time, rapid eye movement (REM) sleep time, and sleep onset latency through polysomnography [44], but the present study did not. In the future, it is also necessary to investigate the association between such objective sleep parameters and PA for firefighters. Fourth, although this study revealed that get-ting enough rest after LTPA was an important factor affecting insomnia, there was no clear definition of enough rest. This portion of the survey was conducted subjectively, with yes or no answers. Further studies involving a clear definition of enough rest after LTPA, at what time during the day the subjects did LTPA, and how long they rested after LTPA are needed.

Despite the above limitations, the advantages of this study are as follow: To the best of the authors’ knowledge, this study is the first one studying the association between LTPA-related characteristics and insomnia according to the OPA degree. Previous studies have only examined the effects of OPA or LTPA on insomnia, respectively. We have elucidated how the two categories of PA affect insomnia. One contribution of this study is the basic data that can suggest the appropriate LTPA to high OPA groups by analyzing how LTPA-related characteristics, such as execution of LTPA, the intensity of LTPA, and getting enough rest after LTPA, affect insomnia according to the OPA degree. Another advantage of this study is that we analyzed data, adjusting for as many covariates as possible related to insomnia, such as socioeconomic, demographic, psychosocial, and organizational characteristics. Therefore, the results of this study can be used to suggest a method for proper LTPA in the high OPA group, including firefighters, police officers, and soldiers.

## 5. Conclusions

This study found that the prevalence and risk of insomnia was higher in the group with high OPA than in the group with low OPA, regardless of LTPA. In addition, the risk of insomnia was significantly lower in the group partaking in LTPA and the group that was getting enough rest after LTPA, regardless of the OPA degree. Finally, in the group with high OPA, subjects doing high-intensity LTPA had a significantly higher risk of insomnia compared to those doing low-intensity LTPA. Therefore, the intensity of exercise should be considered when prescribing exercise to groups with insomnia and high OPA, such as firefighters, police officers, and soldiers.

## Figures and Tables

**Table 1:** Sociodemographic, organizational, and psychological characteristics of the study subjects.

Variable	Total	Normal (ISI < 15)	Insomnia (ISI ≥ 15)	*p*-Value
Total	9788 (100%)	8898 (90.9%)	890 (9.1%)	
Sex				
Male	9724 (93.2%)	8311 (91.1%)	813 (8.9%)	0.020 **
Female	664 (6.8%)	587 (88.4%)	77 (11.6%)	
Age (years) ^a^				
Average	39.58 ± 8.65	39.45 ± 8.63	40.82 ± 8.77	<0.001 *
20–29	1334 (13.6%)	1240 (93.0%)	94 (7.0%)	<0.001 **
30–39	3965 (40.5%)	3626 (91.5%)	339 (8.5%)	
40–49	2865 (29.3%)	2591 (90.4%)	274 (9.6%)	
≥50	1624 (16.6%)	1441 (88.7%)	183 (11.3%)	
BMI (kg/m^2^)				
Average	24.27 ± 2.54	24.27 ± 2.53	24.19 ± 2.65	0.338 *
<25	6233 (63.7%)	5653 (90.7%)	580 (9.3%)	0.333 **
≥25	3555 (36.3%)	3245 (91.3%)	310 (8.7%)	
Education				
High school	2130 (21.8%)	1937 (90.9%)	193 (9.1%)	0.296 **
College	3001 (30.7%)	2747 (91.5%)	254 (8.5%)	
University or graduate school	4657 (47.6%)	4214 (90.5%)	443 (9.5%)	
Marital status ^b^				
Unmarried	2777 (28.4%)	2564 (92.3%)	213 (7.7%)	0.001 **
Married	6858 (70.1%)	6204 (90.5%)	654 (9.5%)	
Others ^c^	153 (1.6%)	130 (85.0%)	23 (15.0%)	
Monthly income (×1000 KRW) ^d^				
<3000	3518 (35.9%)	3247 (92.3%)	271 (7.7%)	0.001 **
3000–4999	4768 (48.7%)	4307 (90.3%)	461 (9.7%)	
≥5000	1502 (15.3%)	1344 (89.5%)	158 (10.5%)	
Smoking status				
Never	3695 (37.8%)	3347 (90.6%)	348 (9.4%)	0.058 **
Ex	3318 (33.9%)	2998 (90.4%)	320 (9.6%)	
Current	2775 (38.4%)	2553 (92.0%)	222 (8.0%)	
Alcohol consumption ^e^				
None or social	7771 (79.4%)	7092 (91.3%)	679 (8.7%)	0.016 **
Moderate to heavy	2017 (20.6%)	1806 (89.5%)	211 (10.5%)	
Caffeine intake ^f^				
None	1514 (15.5%)	1346 (88.9%)	168 (11.1%)	0.001 **
0–2 cups	4933 (50.4%)	4531 (91.9%)	402 (8.1%)	
>2 cups	3341 (34.1%)	3021 (90.4%)	320 (9.6%)	
Type of job ^g^				
Office work	828 (8.5%)	778 (94.0%)	50 (6.0%)	0.008 **
Fire suppression	3145 (32.1%)	2834 (90.1%)	311 (9.9%)	
EMS/rescue	3523 (36.0%)	3202 (90.9%)	321 (9.1%)	
Others	2292 (23.4%)	2084 (90.9%)	208 (9.1%)	
Flexible	3622 (37.0%)	3337 (92.1%)	285 (7.9%)	0.001 **
Non-flexible	6166 (63.0%)	5561 (90.2%)	605 (9.8%)	
Yes	5751 (58.8%)	5269 (91.6%)	482 (8.4%)	0.003 **
No	4037 (41.2%)	3629 (89.9%)	408 (10.1%)	
No	9406 (96.1%)	8738 (92.9%)	668 (7.1%)	<0.001 **
Yes	382 (3.9%)	160 (41.9%)	222 (58.1%)	
No	9497 (97.0%)	8786 (92.5%)	711 (7.5%)	<0.001 **
Yes	291 (3.0%)	112 (38.5%)	179 (61.5%)	
PTSD				
No	8826 (90.2%)	8257 (93.6%)	569 (6.4%)	<0.001 **
Yes	962 (9.8%)	641 (66.6%)	321 (33.4%)	

Data are shown as no. (estimated percentage) for categorical variables and as the mean ± standard error for continuous variables; * the *p*-value by independent two-sample *t*-test; ** the *p*-value by χ^2^ test; ^a^ the distribution of insomnia was significantly different between age groups “20–29” and “40–49,” between age groups “20–29” and “≥50,” and between age groups “30–39” and “≥50” after Bonferroni correction; ^b^ The distribution of insomnia was significantly different between the groups “unmarried” and “married” and between the groups “unmarried” and “others” after Bonferroni correction; ^c^ those who are divorced, widowed, or separated; ^d^ the distribution of insomnia was significantly different between the groups with their monthly income “<3000” and “3000–4999” after Bonferroni correction; ^e^ those who drink ≥ 7 glasses of soju (5 glasses for females) twice a week were labelled as moderate to heavy drinkers; ^f^ the distribution of insomnia was significantly different between the groups who noted their caffeine intake as “none” and “0–2 cups” after Bonferroni correction; ^g^ the distribution of insomnia was significantly different between the various job groups “office work” and “fire suppression”, between the groups “office work” and “EMS/rescue”, and between the groups “office work” and “others” after Bonferroni correction; BMI = body mass index; KRW = Korean Won; PTSD = post-traumatic stress disorder; PHQ-9 = Patient Health Questionnaire-9; GAD-7 = Generalized Anxiety Disorder-7; PC-PTSD = Primary Care PTSD Screen.

**Table 2:** Occupational physical activity (OPA), and leisure-time physical activity (LTPA)-related characteristics of the study subjects.

Variable	Total	Normal (ISI < 15)	Insomnia (ISI ≥ 15)	*p*-Value *
Subjective OPA				
Low	3021 (30.9%)	2882 (95.4%)	139 (4.6%)	<0.001
High	6767 (69.1%)	6016 (88.9%)	751 (11.1%)	
FoJL				
None or little	5781 (59.1%)	5479 (94.8%)	302 (5.2%)	<0.001
Moderate to high	4007 (40.9%)	3419 (85.3%)	588 (14.7%)	
PSUR				
<80%	4561 (46.6%)	4239 (92.9%)	322 (7.1%)	<0.001
≥80%	5227 (53.4%)	4659 (89.1%)	568 (10.9%)	
Objective OPA				
Low	3191 (32.6%)	2949 (92.4%)	242 (7.6%)	<0.001
High	6597 (67.4%)	5949 (90.2%)	648 (9.8%)	
Work schedule				
Day work	1007 (10.3%)	930 (92.4%)	77 (7.6%)	0.092
Shift work	8781 (89.7%)	7968 (90.7%)	813 (9.3%)	
Frequency of occupational activities, per week				
<5	2883 (29.5%)	2667 (92.5%)	216 (7.5%)	<0.001
≥5	6905 (70.5%)	6231 (90.2%)	674 (9.8%)	
Frequency of off-duty work, per month				
<3	8669 (88.6%)	7902 (91.2%)	767 (8.8%)	0.019
≥3	1119 (11.4%)	996 (89.0%)	123 (11.0%)	
Execution of LTPA				
No	2101 (21.5%)	1852 (88.1%)	249 (11.9%)	<0.001
Yes	7687 (78.5%)	7046 (91.7%)	641 (8.3%)	
Type of LTPA				
Does not perform aerobic PA	797 (10.4%)	723 (90.7%)	74 (9.3%)	0.308
Performs aerobic PA	6890 (89.6%)	6323 (91.8%)	567 (8.2%)	
Intensity of LTPA				
Low	5763 (75.0%)	5318 (92.3%)	445 (7.7%)	0.001
High	1924 (25.0%)	1728 (89.8%)	196 (10.2%)	
Sufficient rest after LTPA				
No	974 (12.7%)	788 (80.9%)	186 (19.1%)	<0.001
Yes	6713 (87.3%)	6258 (93.2%)	455 (6.8%)	

* The *p*-value by χ^2^ test; ISI = Insomnia Severity Index; OPA = occupational physical activity; FoJL = feeling of job loading; PSUR = physical strength utilization rate; PA = physical activity; LTPA = leisure time physical activity.

**Table 3:** Association between occupational physical activity (OPA), leisure time physical activity (LTPA), and insomnia. (OR = odds ratio; CI = confidence interval).

Variables		n	Cases	Crude		Adjusted ^a^	
				OR	95% CI	OR	95% CI
**OPA-Related Characteristics**							
Subjective OPA							
Low		3021	139 (4.6%)	1.00	Ref	1.00	Ref
High		6767	751 (11.1%)	2.59	2.15–3.12	2.12	1.73–2.59
	FoJL						
	None or little	5781	302 (5.2%)	1.00	Ref	1.00	Ref
	Moderate to high	4007	588 (14.7%)	3.12	2.70–3.61	2.42	2.06–2.83
	PSUR						
	<80%	4561	322 (7.1%)	1.00	Ref	1.00	Ref
	≥80%	5227	568 (10.9%)	1.61	1.39–1.85	1.42	1.21–1.66
Objective OPA							
Low		3191	242 (7.6%)	1.00	Ref	1.00	Ref
High		6597	648 (9.8%)	1.33	1.14–1.55	1.19	0.99–1.42
	Work schedule						
	Day work	1007	77 (7.6%)	1.00	Ref	1.00	Ref
	Shift work	8781	813 (9.3%)	1.23	0.97–1.57	0.93	0.62–1.37
	Frequency of occupational activities						
	<5/week	2883	216 (7.5%)	1.00	Ref	1.00	Ref
	≥5/week	6905	674 (9.8%)	1.34	1.14–1.57	1.20	1.00–1.43
	Frequency of off-duty work						
	<3/month	8669	767 (8.8%)	1.00	Ref	1.00	Ref
	≥3/month	1119	123 (11.0%)	1.27	1.04–1.56	1.19	0.95–1.50
**LTPA-Related Characteristics**							
Execution of LTPA							
No		2101	249 (11.9%)	1.00	Ref	1.00	Ref
Yes		7687	641 (8.3%)	0.68	0.58–0.79	0.73	0.61–0.88
	Type of LTPA						
	Does not perform aerobic PA	797	74 (9.3%)	1.00	Ref	1.00	Ref
	Performs aerobic PA	6890	567 (8.2%)	0.88	0.68–1.13	0.88	0.66–1.16
	Intensity of LTPA						
	Low	5763	445 (7.7%)	1.00	Ref	1.00	Ref
	High	1924	196 (10.2%)	1.36	1.14–1.62	1.37	1.13–1.67
	Sufficient rest after LTPA						
	No	974	186 (19.1%)	1.00	Ref	1.00	Ref
	Yes	6713	455 (6.8%)	0.31	0.26–0.37	0.40	0.33–0.50

^a^ Adjusted for sex, age, education, marital status, monthly income, smoking status, alcohol consumption, caffeine intake, type of job (office work vs. others), work schedule flexibility, adequacy of notice time, depression, anxiety, and post-traumatic stress disorder; OR = odds ratio; CI = confidence interval; PA = physical activity; OPA = occupational physical activity; LTPA = leisure time physical activity; FoJL = feeling of job loading; PSUR = physical strength utilization rate.

**Table 4:** Association between leisure time physical activity (LTPA) and insomnia according to subjective occupational physical activity (subjective OPA). [aOR = adjusted odds ratio; CI = confidence interval].

Variables		Low Subjective OPA				High Subjective OPA			
		n	Cases	aOR ^a^	95% CI	n	Cases	aOR ^a^	95% CI
Execution of LTPA									
No		617	38 (6.2%)	1.00	Ref	1,484	211 (14.2%)	1.00	Ref
Yes		2404	101 (4.2%)	0.61	0.40–0.94	5283	540 (10.2%)	0.77	0.62–0.94
	Type of LTPA								
	Do not perform aerobic PA	240	14 (13.9%)	1.00	Ref	557	60 (10.8%)	1.00	Ref
	Perform aerobic PA	2164	87 (4.0%)	0.62	0.33–1.18	4726	480 (10.2%)	0.95	0.69–1.30
	Intensity of LTPA								
	Low	1740	73 (4.2%)	1.00	Ref	4023	372 (9.2%)	1.00	Ref
	High	664	28 (4.2%)	0.96	0.59–1.56	1260	168 (13.3%)	1.52	1.23–1.89
	Sufficient rest after LTPA								
	No	229	27 (11.8%)	1.00	Ref	745	159 (21.3%)	1.00	Ref
	Yes	2175	74 (3.4%)	0.33	0.20–0.56	4538	381 (8.4%)	0.43	0.34–0.55

^a^ Adjusted for sex, age, education, marital status, monthly income, smoking status, alcohol consumption, caffeine intake, type of job (office work vs. others), work schedule flexibility, adequacy of notice time, depression, anxiety, and post-traumatic stress disorder; OR = odds ratio; CI = confidence interval; PA = physical activity; OPA = occupational physical activity; LTPA = leisure time physical activity.

**Table 5:** Association between leisure time physical activity (LTPA) and insomnia according to objective occupational physical activity (objective OPA). [aOR = adjusted odds ratio; CI = confidence interval].

Variables		Low Objective OPA				High Objective OPA			
		n	Cases	aOR ^a^	95% CI	n	Cases	aOR ^a^	95% CI
Execution of LTPA									
No		811	72 (8.9%)	1.00	Ref	1290	177 (13.7%)	1.00	Ref
Yes		2380	170 (7.1%)	0.77	0.55–1.09	5307	471 (8.9%)	0.72	0.58–0.90
	Type of LTPA								
	Do not perform aerobic PA	227	15 (6.6%)	1.00	Ref	570	59 (10.4%)	1.00	Ref
	Perform aerobic PA	2153	155 (7.2%)	1.20	0.65–2.21	4737	412 (8.7%)	0.81	0.59–1.11
	Intensity of LTPA								
	Low	1870	126 (6.7%)	1.00	Ref	3893	319 (8.2%)	1.00	Ref
	High	510	44 (8.6%)	1.17	0.78–1.76	1414	152 (10.7%)	1.44	1.15–1.81
	Sufficient rest after LTPA								
	No	242	38 (15.7%)	1.00	Ref	732	148 (20.2%)	1.00	Ref
	Yes	2138	132 (6.2%)	0.40	0.26–0.62	4575	323 (7.1%)	0.41	0.32–0.52

^a^ Adjusted for sex, age, education, marital status, monthly income, smoking status, alcohol consumption, caffeine intake, type of job (office work vs. others), work schedule flexibility, adequacy of notice time, depression, anxiety, and post-traumatic stress disorder; OR = odds ratio; CI = confidence interval; PA = physical activity; OPA = occupational physical activity; LTPA = leisure time physical activity.

## Supplementary Materials

The following are available online at https://www.mdpi.com/1660-4601/17/15/5397/s1, Table S1: Association between leisure time physical activity (LTPA) and insomnia according to subjective occupational physical activity (subjective OPA) in the group without psychological symptoms (n = 8578), Table S2: Association between leisure time physical activity (LTPA) and insomnia according to objective occupational physical activity (objective OPA) in the group without psychological symptoms.

## Abbreviations

PA	physical activity
ISI	Insomnia Severity Index
PSQI	Pittsburgh Sleep Quality Index
LTPA	leisure time physical activity
OPA	occupational physical activity
FoJL	feeling of job loading
PSUR	physical strength utilization rate
BMI	body mass index
KRW	Korean Won
EMS	emergency medical service
PTSD	post-traumatic stress disorder
PHQ-9	Patient Health Questionnaire-9
GAD-7	Generalized Anxiety Disorder-7
PC-PTSD	Primary Care-PTSD Screen for DSM-5
OR	odds ratio
CI	confidence interval

## References

[1] LeBlanc M., Merette C., Savard J., Ivers H., Baillargeon L., Morin C.M. (2009). Incidence and risk factors of insomnia in a population-based sample. Sleep.

[2] Ohayon M.M. (2002). Epidemiology of insomnia: What we know and what we still need to learn. Sleep Med. Rev..

[3] Hartescu I., Morgan K. (2019). Regular physical activity and insomnia: An international perspective. J. Sleep Res..

[4] Skarupke C., Schlack R., Lange K., Goerke M., Dueck A., Thome J., Szagun B., Cohrs S. (2017). Insomnia complaints and substance use in german adolescents: Did we underestimate the role of coffee consumption? Results of the kiggs study. J. Neural Transm..

[5] Foley D.J., Monjan A., Simonsick E.M., Wallace R.B., Blazer D.G. (1999). Incidence and remission of insomnia among elderly adults: An epidemiologic study of 6,800 persons over three years. Sleep.

[6] Youngstedt S.D., Kline C.E. (2006). Epidemiology of exercise and sleep. Sleep Biol. Rhythm..

[7] Loprinzi P.D., Cardinal B.J. (2011). Association between objectively-measured physical activity and sleep, nhanes 2005–2006. Ment. Health Phys. Act..

[8] Flausino N.H., Prado J.M.D., de Queiroz S.S., Tufik S., de Mello M.T. (2012). Physical exercise performed before bedtime improves the sleep pattern of healthy young good sleepers. Psychophysiology.

[9] Chennaoui M., Arnal P.J., Sauvet F., Leger D. (2015). Sleep and exercise: A reciprocal issue?. Sleep Med. Rev..

[10] Kredlow M.A., Capozzoli M.C., Hearon B.A., Calkins A.W., Otto M.W. (2015). The effects of physical activity on sleep: A meta-analytic review. J. Behav. Med..

[11] Skarpsno E.S., Mork P.J., Nilsen T.I.L., Jorgensen M.B., Holtermann A. (2018). Objectively measured occupational and leisure-time physical activity: Cross-sectional associations with sleep problems. Scand. J. Work Environ. Health.

[12] Wilson J.R., Sharples S. (2015). Evaluation of Human Work.

[13] Lee S.J., Moon M.K., Choi W.J., Jang T.W. (2019). Exercise and cardiovascular load in workers with high occupational physical activity. Arch. Environ. Occup. Health.

[14] Holtermann A., Marott J.L., Gyntelberg F., Sogaard K., Mortensen O.S., Prescott E., Schnohr P. (2016). Self-reported occupational physical activity and cardiorespiratory fitness: Importance for cardiovascular disease and all-cause mortality. Scand. J. Work Environ. Health.

[15] Ahn Y.S. (2018). The Study on Establishment of Activity System and Ensuring Business Continuity of Firefighters.

[16] U.S. Department of Labor (1991). Employment and Training Administration. The Revised Handbook for Analyzing Jobs.

[17] Ainsworth B.E., Haskell W.L., Herrmann S.D., Meckes N., Bassett D.R., Tudor-Locke C., Greer J.L., Vezina J., Whitt-Glover M.C., Leon A.S. (2011). 2011 compendium of physical activities: A second update of codes and met values. Med. Sci. Sports Exerc..

[18] Park J.T. (2014). Analysis of Fire Fighter’s Work Intensity of Scene Activities and Their Physiological Changes.

[19] Pawlak R., Clasey J.L., Palmer T., Symons T.B., Abel M.G. (2015). The effect of a novel tactical training program on physical fitness and occupational performance in firefighters. J. Strength Cond. Res..

[20] Choi S.J., Song P., Suh S., Joo E.Y., Lee S.I. (2020). Insomnia symptoms and mood disturbances in shift workers with different chronotypes and working schedules. J. Clin. Neurol..

[21] Jang T.W., Jeong K.S., Ahn Y.S., Choi K.S. (2020). The relationship between the pattern of shift work and sleep disturbances in korean firefighters. Int. Arch. Occup. Environ. Health.

[22] U.S. Department of Health and Human Services (2018). Physical Activity Guidelines for Americans.

[23] Bastien C.H., Vallieres A., Morin C.M. (2001). Validation of the insomnia severity index as an outcome measure for insomnia research. Sleep Med..

[24] Kroenke K., Spitzer R.L., Williams J.B. (2001). The phq-9: Validity of a brief depression severity measure. J. Gen. Intern. Med..

[25] Spitzer R.L., Kroenke K., Williams J.B., Lowe B. (2006). A brief measure for assessing generalized anxiety disorder: The gad-7. Arch. Intern. Med..

[26] Spoont M.R., Williams J.W., Kehle-Forbes S., Nieuwsma J.A., Mann-Wrobel M.C., Gross R. (2015). Does this patient have posttraumatic stress disorder? Rational clinical examination systematic review. JAMA.

[27] Jirathananuwat A., Pongpirul K. (2017). Promoting physical activity in the workplace: A systematic meta-review. J. Occup. Health..

[28] Thompson J.F., Severson R.L., Rosecrance J.C. (2018). Occupational physical activity in brewery and office workers. J. Occup. Environ. Hyg..

[29] Zimring C., Joseph A., Nicoll G.L., Tsepas S. (2005). Influences of building design and site design on physical activity: Research and invervention opportunities. Am. J. Prev. Med..

[30] Sawyer A., Smith L., Ucci M., Jones R., Marmot A., Fisher A. (2017). Perceived office environments and occupational physical activity in office-based workers. Occup. Med. (Lond.).

[31] Ahn Y.S. (2011). The Analysis of Risk Factors Related Health and Safety at Disasters and Development of Special Medical Health Examination System for Firefighters.

[32] Salo P., Ala-Mursula L., Rod N.H., Tucker P., Pentti J., Kivimaki M., Vahtera J. (2014). Work time control and sleep disturbances: Prospective cohort study of finnish public sector employees. Sleep.

[33] Boivin D.B., Boudreau P. (2014). Impacts of shift work on sleep and circadian rhythms. Pathol. Biol. Paris.

[34] Lee C.G., Middlestadt S.E., Park S., Kwon J., Noh K., Seo D.I., Song W., Park J.J., Lee H.J., Kang H.J. (2020). Predicting voluntary exercise training among korean firefighters: Using elicitation study and the theory of planned behavior. Int. J. Environ. Res. Public Health.

[35] Park S., Kwon J., Noh K., Lee C.G., Song W., Park J.J., Lee H.J., Seo D.I., Kang H.J., Ahn Y.S. (2020). Relationship between shift type and voluntary exercise training in south korean firefighters. Int. J. Environ. Res. Public Health.

[36] Warburton D.E., Nicol C.W., Bredin S.S. (2006). Health benefits of physical activity: The evidence. CMAJ.

[37] Buxton O.M., L’Hermite-Baleriaux M., Hirschfeld U., Cauter E. (1997). Acute and delayed effects of exercise on human melatonin secretion. J. Biol. Rhythms.

[38] Adrien J., Billiard M. (2003). Neurology of the sleep-wake cycle. Sleep: Physiology, Investigations and Medicine.

[39] Uusitalo A.L., Valkonen-Korhonen M., Helenius P., Vanninen E., Bergstrom K.A., Kuikka J.T. (2004). Abnormal serotonin reuptake in an overtrained, insomnic and depressed team athlete. Int. J. Sports Med..

[40] Jansson-Frojmark M., Lindblom K. (2008). A bidirectional relationship between anxiety and depression, and insomnia? A prospective study in the general population. J. Psychosom. Res..

[41] Costa G., Sartori S., Akerstedt T. (2006). Influence of flexibility and variability of working hours on health and well-being. Chronobiol. Int..

[42] Takahashi M., Iwasaki K., Sasaki T., Kubo T., Mori I., Otsuka Y. (2011). Worktime control-dependent reductions in fatigue, sleep problems, and depression. Appl. Ergon..

[43] Kubo T., Takahashi M., Togo F., Liu X., Shimazu A., Tanaka K., Takaya M. (2013). Effects on employees of controlling working hours and working schedules. Occup. Med. Lond..

[44] Mesas A.E., Hagen E.W., Peppard P.E. (2018). The bidirectional association between physical activity and sleep in middle-aged and older adults: A prospective study based on polysomnography. Sleep.

