# Supplementary material for: Effects of Occupational and Leisure-Time Physical Activities on Insomnia in Korean Firefighters
Source: Int J Environ Res Public Health. 2020 Jul 27;17(15):5397. doi: 10.3390/ijerph17155397 (PMC7432718; doi:10.3390/ijerph17155397)
Supplement: Supplementary file 1 [file ijerph-17-05397-s001.pdf]

Supplementary Materials

Table S1. Association between leisure time physical activity (LTPA) and insomnia according to subjective occupational physical activity (subjective OPA) in the group without psychological symptoms (n= 8,578). [aOR = adjusted odds ratio; CI = confidence interval].

Variables	Low Subjective OPA				High Subjective OPA			
	n	Cases	aOR [a]	95% CI	n	Cases	aOR [a]	95% CI
Do LTPA								
No	553	22 (4.0%)	1.00	Ref	1214	94 (7.7%)	1.00	Ref
Yes	2221	64 (2.9%)	0.617	0.376–1.045	4590	281 (6.1%)	0.720	0.560–0.925
Kind of LTPA								
Do not perform aerobic PA	217	7 (3.2%)	1.00	Ref	475	34 (7.2%)	1.00	Ref
Perform aerobic PA	2004	57 (2.8%)	0.748	0.332–1.687	4115	247 (6.0%)	0.804	0.552–1.170
Intensity of LTPA								
Low	1604	50 (3.1%)	1.00	Ref	3511	198 (5.6%)	1.00	Ref
High	617	14 (2.3%)	0.732	0.396–1.353	1079	83 (7.7%)	1.388	1.059–1.819
Enough rest after LTPA								
No	201	16 (8.0%)	1.00	Ref	577	66 (11.4%)	1.00	Ref
Yes	2020	48 (2.4%)	0.287	0.158–0.522	4013	215 (5.4%)	0.440	0.327–0.592

[a] adjusted for sex, age, education, marital status, monthly income, smoking status, alcohol consumption, caffeine intake, type of job (office work vs. others), work schedule flexibility, adequacy of notice time; OR = odds ratio; CI = confidence interval; PA = physical activity; OPA = occupational physical activity; LTPA = leisure time physical activity.

Table S2. Association between leisure time physical activity (LTPA) and insomnia according to objective occupational physical activity (objective OPA) in the group without psychological symptoms (n = 8,578). [aOR = adjusted odds ratio; CI = confidence interval].

Variables	Low Objective OPA				High Objective OPA			
	n	Cases	aOR [a]	95% CI	n	Cases	aOR [a]	95% CI
Do LTPA								
No	693	33 (4.8%)	1.00	Ref	1074	83 (7.7%)	1.00	Ref
Yes	2138	97 (4.5%)	0.751	0.490–1.152	4673	248 (5.3%)	0.667	0.512–0.869
Kind of LTPA								
Do not perform aerobic PA	199	12 (6.0%)	1.00	Ref	493	29 (5.9%)	1.00	Ref
Perform aerobic PA	1939	85 (4.4%)	0.661	0.350–1.248	4180	219 (5.2%)	0.855	0.571–1.280
Intensity of LTPA								
Low	1687	77 (4.6%)	1.00	Ref	3428	171 (5.0%)	1.00	Ref
High	451	20 (4.4%)	1.034	0.620–1.727	1245	77 (6.2%)	1.267	0.955–1.680
Enough rest after LTPA								
No	204	22 (10.8%)	1.00	Ref	574	60 (10.5%)	1.00	Ref
Yes	1934	75 (3.9%)	0.320	0.191–0.536	4099	188 (4.6%)	0.420	0.308–0.573

[a] adjusted for sex, age, education, marital status, monthly income, smoking status, alcohol consumption, caffeine intake, type of job (office work vs. others), work schedule flexibility, adequacy of notice time; OR = odds ratio; CI = confidence interval; PA = physical activity; OPA = occupational physical activity; LTPA = leisure time physical activity.
